# The closest lineage of Archaeplastida is revealed by phylogenomics analyses that include *Microheliella maris*

**DOI:** 10.1098/rsob.210376

**Published:** 2022-04-13

**Authors:** Euki Yazaki, Akinori Yabuki, Ayaka Imaizumi, Keitaro Kume, Tetsuo Hashimoto, Yuji Inagaki

**Affiliations:** ^1^ RIKEN iTHEMS, Wako, Saitama 351-0198, Japan; ^2^ Japan Agency for Marine-Earth Science and Technology, Yokosuka, Kanagawa 237-0061, Japan; ^3^ College of Biological Sciences, University of Tsukuba, Tsukuba, Ibaraki 305-8572, Japan; ^4^ Faculty of Medicine, University of Tsukuba, Tsukuba, Ibaraki 305-8572, Japan; ^5^ Faculty of Life and Environmental Sciences, University of Tsukuba, Tsukuba, Ibaraki 305-8572, Japan; ^6^ Graduate School of Life and Environmental Sciences, University of Tsukuba, Tsukuba, Ibaraki 305-8572, Japan; ^7^ Center for Computational Sciences, University of Tsukuba, Tsukuba, Ibaraki 305-8572, Japan

**Keywords:** phylogenetic artefacts, Cryptista, Cryptophyceae, Goniomonadea, global eukaryotic phylogeny

## Abstract

By clarifying the phylogenetic positions of ‘orphan’ protists (unicellular micro-eukaryotes with no affinity to extant lineages), we may uncover the novel affiliation between two (or more) major lineages in eukaryotes. *Microheliella maris* was an orphan protist, which failed to be placed within the previously described lineages by pioneering phylogenetic analyses. In this study, we analysed a 319-gene alignment and demonstrated that *M. maris* represents a basal lineage of one of the major eukaryotic lineages, Cryptista. We here propose a new clade name ‘Pancryptista’ for Cryptista plus *M. maris*. The 319-gene analyses also indicated that *M. maris* is a key taxon to recover the monophyly of Archaeplastida and the sister relationship between Archaeplastida and Pancryptista, which is collectively called ‘CAM clade’ here. Significantly, Cryptophyceae tend to be attracted to Rhodophyta depending on the taxon sampling (ex., in the absence of *M. maris* and Rhodelphidia) and the particular phylogenetic ‘signal’ most likely hindered the stable recovery of the monophyly of Archaeplastida in previous studies.

## Graphical abstract

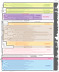

## 1. Background

Our understanding of the evolutionary relationship among major eukaryotic groups has been progressed constantly. The foundation of the tree of eukaryotes was developed initially based on the combination of morphological characteristics (including those on the ultrastructural level) and molecular phylogenetic analyses of a single or few marker genes [1–3]. In recent years, ‘phylogenomic’ analyses—phylogenetic analyses of large-scale multigene alignments, particularly those comprising hundreds of genes—were often conducted to reconstruct deep splits in the tree of eukaryotes with high statistical support [4–7]. For instance, recent phylogenomic analyses have constantly reconstructed the clade of stramenopiles, Alveolata, and Rhizaria (SAR clade) [8], that of Opisthokonta, Amoebozoa, Breviatea and Apusomonadida (Amorphea) [9], and that of Collodictyonidae, Rigifilida and *Mantamonas* (CRuMs) [10].

There are many unicellular micro-eukaryotic lineages of which phylogenetic positions remain uncertain (‘orphan’ lineages). Some of the current orphan lineages most likely represent as-yet-unknown portions of the diversity of eukaryotes and hold clues to resolve the eukaryotic evolution. Prior to DNA sequencing experiments gaining in popularity in phylogenetic/taxonomic studies, diverse eukaryotes were isolated from the natural environments and examined by microscopes. If the morphological characteristics of the eukaryotes of interest showed no clear affinity to any other eukaryotes, their phylogenetic affiliations remained uncertain [11–15]. The analyses of small subunit ribosomal DNA (SSU rDNA)—one of the most popular gene markers for organismal phylogeny—succeeded in finding the phylogenetic homes of many lineages, of which morphological information was insufficient to resolve their phylogenetic affiliations [16–19]. More recently, orphan lineages, as well as newly found eukaryotes have been subjected to phylogenomic analyses [8,9,20–27].

Phylogenomic analyses are not always valid for elucidating the phylogenetic positions of all of the orphan lineages recognized to date. For instance, the positions of Malawimonadida [28], Ancyromonadida [10], Hemimastigophora [29], *Ancoracysta twista* [30] and *Microheliella maris* [31] could not be clarified even after phylogenomic analyses. The pioneering studies might have failed to clarify the phylogenetic positions of the orphan lineages listed above due to insufficient data and/or taxon sampling in the alignments and various forms of systematic artefacts in tree reconstruction (e.g. long branch attraction or LBA [32]). However, there is a possibility that some of the orphan lineages are genuine deep branches that are critical to resolving the backbone of the tree of eukaryotes. In this study, we attempted to clarify the phylogenetic position of *M. maris* by analysing a new phylogenomic alignment. *Microheliella maris* was originally described as a member of the phylum Heliozoa based on the shared morphological similarities (e.g. the radiating axopodia with tiny granules and the centroplast) [33]. Cavalier-Smith *et al*. [31] then examined the phylogenetic position of *M. maris* by analysing the alignment comprising 187 genes. Nevertheless, *M. maris* is still regarded as one of the orphan eukaryotes [34], as the choice of the methods for tree reconstruction and taxon sampling affected largely the position of this eukaryote in the 187-gene phylogeny [31].

We here reassessed the phylogenetic position of *M. maris* by analysing a new phylogenomic alignment comprising 319 genes (88 592 amino acid positions in total). The 319-gene phylogeny placed *M. maris* at the base of the Cryptista clade with high statistical support, suggesting that this eukaryote holds keys to understanding the early evolution of Cryptista as well as Diaphoretickes. Indeed, we further demonstrated that *M. maris* and Rhodelphidia, which occupy the basal position of Cryptista and that of Rhodophyta, respectively, suppress the erroneous ‘signal’ attracting Cryptophyceae and Rhodophyta to each other and contribute to recovering (i) the monophyly of Archaeplastida and (ii) the sister relationship between Archaeplastida and the clade of Cryptista plus *M. maris*. Finally, we explored the biological ground for the phylogenetic artefact uniting Cryptophyceae and Rhodophyta together.

## 2. Methods

### 2.1. Cell culturing and RNA-seq analysis

We generated the RNA-seq data from *M. maris* and *Hemiarma marina*, a species of Goniomonadea, in this study. The culture of *M. maris* (studied in Yabuki *et al*. [33]) and that of *H. marina* (established in Shiratori and Ishida [35]) have been kept in the laboratory and were used in this study. The harvested cells of both organisms were subjected to RNA extraction using TRIzol (Life Technologies) by following the manufacturer's instructions. We shipped the two RNA samples to a biotech company (Hokkaido System Science) for cDNA library construction from the poly-A-tailed RNAs followed by sequencing using the Illumina Hi-seq 2000 platform. For *M. maris*, 1.6 × 10^7^ paired-end 100 bp reads (1.6 Gb in total) were obtained and then assembled into 30 305 unique contigs by TRINITY v. 2.8.4 [36,37]. For *H. marina*, we obtained 1.9 × 10^7^ paired-end 100 bp reads (1.9 Gb in total) and assembled them into 41 539 unique contigs by TRINITY v. 2.8.4 [36,37].

### 2.2. Global eukaryotic phylogeny

To elucidate the phylogenetic position of *M. maris*, we prepared a phylogenomic alignment by updating an existing dataset comprising 351 genes [29]. For each of the 351 genes, we added the homologous sequences retrieved by TBLASTN (*E*-value cut-off was set to 10^−30^) from the transcriptomic data newly generated from *M. maris* and *H. marina* in this study (see above), as well as other eukaryotes that were absent in the original data [29], such as *Marophrys* sp. SRT127 [38], two species of Rhodelphidia (i.e. *Rhodelphis limneticus* and *R. marinus*) [26], and *Ancoracysta twista* [30]. Individual single-gene alignments were aligned by MAFFT v. 7.205 [39,40] with the L-INS-i algorithm followed by manual correction and exclusion of ambiguously aligned positions. Each of the single-gene alignments was subjected to a preliminary phylogenetic analysis using FASTTREE v. 2.1 [41,42] under the LG + *Γ* model. The resultant approximately maximum-likelihood trees with SH-like local supports were inspected to identify the alignments bearing aberrant phylogenetic signal that disagreed strongly with any of a set of well-established monophyletic assemblages in the tree of eukaryotes, namely Opisthokonta, Amoebozoa, Alveolata, stramenopiles, Rhizaria, Rhodophyta, Chloroplastida, Glaucophyta, Haptophyta, Cryptista, Jakobida, Euglenozoa, Heterolobosea, Diplomonadida, Parabasalia and Malawimonadida. A total of 32 out of the 351 single-gene alignments were found to violate the above-mentioned criteria and were excluded from the phylogenomic analyses described below. The remaining 319 single-gene alignments (electronic supplementary material, table S1) were concatenated into a single phylogenomic alignment containing 82 taxa with 88 592 unambiguously aligned amino acid positions. The coverage for each single-gene alignment is summarized in electronic supplementary material, table S1.

We first subjected the final alignment comprising 319 genes from 82 taxa (GlobE alignment) to the maximum-likelihood (ML) method by IQ-TREE v. 1.6.12 [43] with the LG + *Γ* + F + C60 model [44]. The robustness of the ML phylogenetic tree was evaluated with a non-parametric ML bootstrap analysis with the LG + *Γ* + F + C20 + PMSF (posterior mean site frequencies) model (100 replicates). The ML tree inferred with the LG + *Γ* + F + C60 model was used as the guide tree for the bootstrap analysis incorporating PMSF. We also conducted Bayesian phylogenetic analysis with the CAT + GTR model using PHYLOBAYES-mpi v. 1.8a [45,46]. In this analysis, two MCMC runs were run for 5000 cycles with ‘burn-in’ of 1250. The consensus tree with branch lengths and Bayesian posterior probabilities (BPPs) were calculated from the remaining trees.

We evaluated the contribution of fast-evolving positions in the GlobE alignment to the position of *M. maris*. Substitution rates of individual alignment positions were calculated over the ML tree by IQ-TREE v. 1.6.12 [43] and top 20%, 40%, 60% and 80% fastest-evolving positions were then removed from the original alignment. The processed alignments were then subjected to the ML bootstrap analysis with the UFBOOT approximation [47] (1000 replicates) by using IQ-TREE v. 1.6.12 [43] with the LG + *Γ* + F model. Henceforth, the alignment modification and the following ML bootstrap analyses are designated as ‘FPR (fast-evolving position removal)’ analysis.

We also examined the impact of the sampling of the genes in the GlobE alignment on the position of *M. maris* by ‘RGS (random gene sampling)’ analyses described below [48]. From the 319 genes in the GlobE alignment, 50 genes were randomly sampled and concatenated into a single alignment (‘rs50g’ alignment). The above procedure was repeated 50 times to obtain 50 of rs50g alignments. Likewise, we prepared (i) 50 of ‘rs100g’ alignments comprising 100 randomly sampled genes, (ii) 10 of ‘rs150g’ alignments comprising 150 randomly sampled genes and (iii) 10 of ‘rs200g’ alignments comprising 200 randomly sampled genes. The alignments comprising randomly sampled genes were subjected individually to the ML bootstrap analysis with the UFBOOT approximation (1000 replicates) by using IQ-TREE v. 1.6.12 [43] with the LG + *Γ* + F model.

### 2.3. Diaphoretickes phylogeny

To evaluate the impact of the inclusion of *M. maris* to the phylogenetic relationship among the species/lineages in Dipahoretickes, we excluded 22 taxa from the GlobE alignment to generate the second phylogenomic alignment, of which taxa were mostly members of Diaphoretickes. Note that the number of genes remained the same between the GlobE and the second, ‘Diaph’ alignments. The Diaph alignment was subjected to both ML and Bayesian phylogenetic analysis under all the same conditions as described above, except that we used the LG + *Γ* + F + C60 + PMSF model for the ML bootstrap analysis with the ML tree inferred with the LG + *Γ* + F + C60 model as the guide tree. Both FPR and RGS analyses (see above) were applied to the Diaph alignment. We also conducted both FPR and RGS analyses after excluding Rhodelphidia and *M. maris* alternatively from the Diaph alignment.

The taxon sampling of the Diaph alignment was further modified by excluding (i) Rhodelphidia and *M. maris*, (ii) Rhodelphidia, *M. maris* and *Palpitomonas bilix*, (iii) Rhodelphidia, *M. maris*, *P. bilix* and Goniomonadea and (iv) Rhodelphidia, *M. maris*, *P. bilix* and Cryptophyceae. We ran RGS analyses of all of the four alignments described above, and the last two were subjected to FPR analyses as well.

## 3. Results and discussion

### 3.1. *Microheliella maris* represents a lineage basal to Cryptista: proposal of ‘Pancryptista’

**Figure 1.**
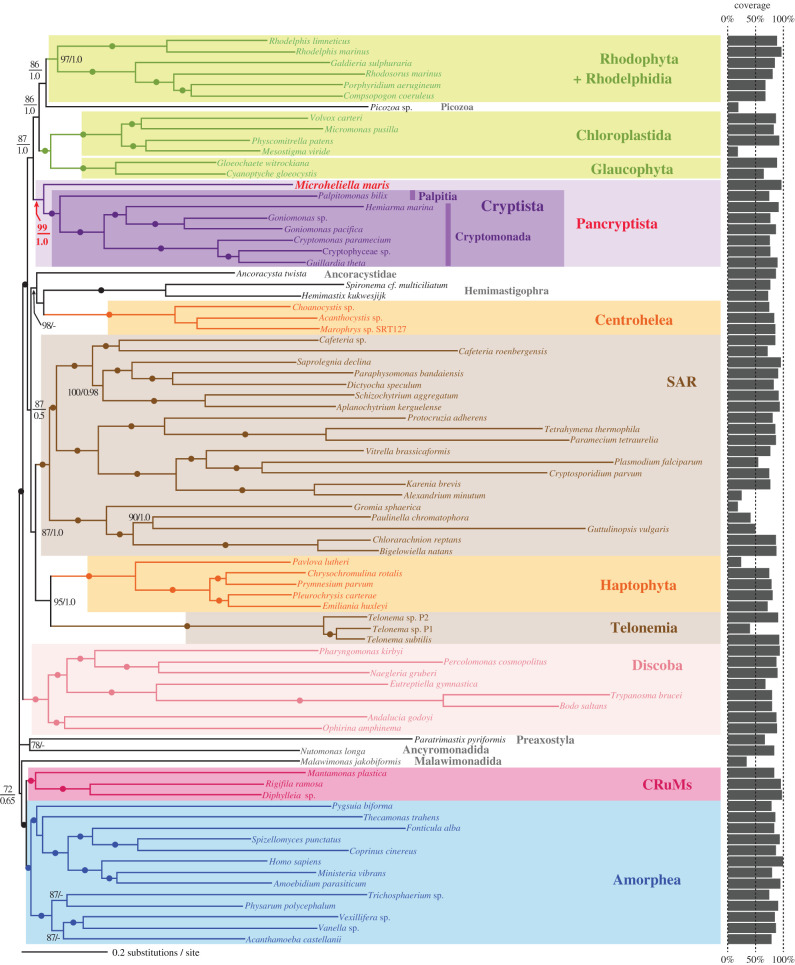
Phylogenetic position of *Microheliella maris* inferred from the GlobE alignment. The tree topology and branch lengths were inferred from the GlobE alignment (319 genes; 88 592 amino acid positions in total) by the maximum-likelihood (ML) methods. Bayesian analysis recovered principally an identical tree topology (electronic supplementary material, figure S1). For each bipartition, the ML bootstrap support values (MLBPs) and Bayesian posterior probabilities (BPPs; if greater than 0.50) are shown. The bipartitions with dots indicate MLBPs of 100% and BPPs of 1.0. The bar graph for each taxon represents the per cent coverage of the amino acid positions in the GlobE alignment.

We analysed a transcriptome-based GlobE alignment consisting of 319 genes sampled from 82 eukaryotes, which represent the major taxonomic assemblages and several orphan taxa/lineages. The GlobE phylogeny recovered the major clades in eukaryotes, such as SAR, Amorphea, CRuMs, Discoba and Cryptista with full statistical support in both ML and Bayesian methods (figure 1; see also electronic supplementary material, figure S1). *Microheliella maris* branched at the base of the Cryptista clade, which comprises *P. bilix*, Goniomonadea including *Hemiarma marina*, and Cryptophyceae, with an MLBP of 99% and a BPP of 1.0. The GlobE alignment includes no data of Kathablepharidacea which is the other cryptistan subgroup, as their available data are extremely low site coverage. However, the lack of Kathablepharidacea most likely had little impact on the phylogenetic position of *M. maris* relative to Cryptista, as far as the alignment includes *P. bilix* which is more basal than Kathablepharidacea in the Cryptista clade [21]. The monophyly of Archaeplastida including Rhodelphidia (*Rhodelphis limneticus* and *R. marinus*) and Picozoa sp., both of which grouped with Rhodophyta, was recovered with an MLBP of 87% and a BPP of 1.0. The intimate affinity of Rhodelphidia and Picozoa to Rhodophyta in the GlobE phylogeny is consistent with the recent phylogenomic studies [26,49]. Neither of the two recently proposed major clades in eukaryotes, T-SAR (Telonemia plus SAR) [24] and Haptista (Centrohelea plus Haptophyta) [22], was reconstructed. Either or both ML and Bayesian phylogenetic analyses failed to give full statistical support to the nodes connecting the lineages/species in Diaphoretickes, namely Archaeplastida, Centrohelea, Haptophyta, Telonemia, SAR and Cryptista plus *M. maris*. Thus, we conclude that the analyses of the GlobE alignment are insufficient to retrace the early evolution of Diaphoretickes with confidence.

We here examined the phylogenetic position of *M. maris* inferred from the GlobE alignment by the progressive removal of fast-evolving positions (FPR analyses). The contribution of fast-evolving positions in the GlobE alignment to the union of *M. maris* and Cryptista is most likely negligible, as the ultrafast bootstrap support values (UFBPs) for the clade comprising *M. maris* and Cryptista stayed 100% until the top 80% fastest-evolving positions were removed (purple line in electronic supplementary material, figure S2a). We detected two conflicting phylogenetic signals regarding the position of *M. maris* relative to the members of Cryptista included in the GlobE alignment, one placing *M. maris* at the base of the Cryptista clade and the other uniting *M. maris* and *P. bilix* directly (red and yellow lines, respectively, in electronic supplementary material, figure S2a). However, the former signal constantly dominated over the latter, regardless of the amount of fast-evolving positions in the alignment. Thus, we conclude that the basal position of *M. maris* to the Cryptista clade in the GlobE phylogeny (figure 1) is free from potential phylogenetic artefacts stemming from fast-evolving positions.

To evaluate the impact of gene sampling on the position of *M. maris* in the GlobE phylogeny, we randomly sampled 50 genes, 100 genes, 150 genes and 200 genes from the 319 genes and concatenated them into ‘rs50g,’ ‘rs100g,’ ‘rs150g,’ and ‘rs200g’ alignments, respectively (note that the taxon sampling remained the same). The UFBPs for the clade comprising *M. maris* and Cryptista calculated from 50 of rs50g alignments and 50 of rs100g alignments distributed from 0 (or nearly 0) to 100% (electronic supplementary material, figure S2b; see also electronic supplementary material, table S2 for the details). The UFBP for the clade of *M. maris* and Cryptista appeared to be less than 40% in the analyses of 12 out of the 50 of rs50g alignments and five out of the 50 of rs100g alignments, albeit the same analyses supported the monophyly of Cryptista with UFBPs of 76–100% (electronic supplementary material, table S2). The above-mentioned results likely reflect the relative abundance of phylogenetic signal for the clade of Cryptista over that for the grouping of *M. maris* and Cryptista together in the 319 genes. Nevertheless, in the analyses of rs150g and rs200g alignments, the UFBP for the grouping of *M. maris* and Cryptista increased. Significantly, the support for the grouping of *M. maris* and Cryptista received UFBPs of 81.3–100% in the analyses of rs200g alignments (the rightmost plot in electronic supplementary material, figure S2b; see also electronic supplementary material, table S2). As observed in FPR analysis of GlobE alignment (electronic supplementary material, figure S2a), rs50g and rs100g alignments appeared to contain two conflicting signals for the phylogenetic position of *M. maris*, one placing *M. maris* at the base of the Cryptista clade and the other uniting *M. maris* and *P. bilix* directly (electronic supplementary material, figure S2c and d). In the analyses of rs150g alignments, the UFBP for the basal position of *M. maris* to the Cryptista clade appeared to be greater than that for the direct union of *M. maris* and *P. bilix* in eight out of the 10 cases (electronic supplementary material, table S2). The same trend was observed in the analyses of rs200g alignments, albeit the signal for the clade grouping *M. maris* and *P. bilix* remained detectable (electronic supplementary material, figure S2d). This series of analyses demonstrated that the greater the number of genes included in the alignment, the greater the UFBP for the basal position of *M. maris* at the Cryptista clade. Based on the results described above, we conclude that the basal position of *M. maris* to the Cryptista clade is genuine, and henceforth designate the clade grouping *M. maris* and the previously known Cryptista as Pancryptista.

### 3.2. The sister relationship between Archaeplastida and Pancryptista: proposal of ‘CAM clade’

**Figure 2.**
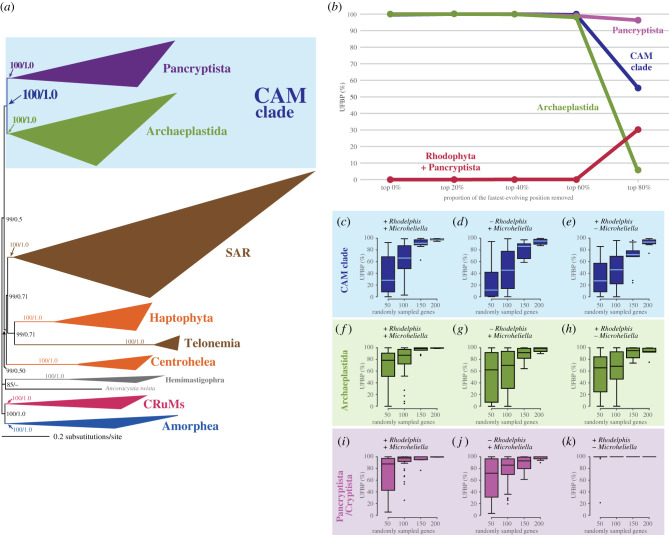
Analyses assessing the impact of *Microheliella maris* and Rhodelphidia on the monophyly of Pancryptista, the monophyly of Archaeplastida and CAM clade. We here define CAM clade as the sister relationship between Pancryptista and Archaeplastida on the top of the monophyly of each of both assemblages. If Pancryptista (or Cryptista) is directly grouped with Rhodophyta (the union of Pancryptista–Rhodophyta disrupts the monophyly of Archaeplastida), we do not consider the clade of Pancryptista/Cryptista, Rhodophyta, Chloroplastida, and Glaucophyta as CAM clade. (*a*) The maximum-likelihood (ML) tree inferred from the Diaph alignment comprising 319 genes (88 592 amino acid positions in total). Clades of closely related taxa are collapsed as triangles. For the detailed ML tree, please refer to electronic supplementary material, figure S2. Bayesian analysis recovered principally an identical tree topology (electronic supplementary material, figure S3). ML bootstrap support values (MLBPs) and Bayesian posterior probabilities (BPPs; if greater than 0.50) are indicated on the bipartitions presented in the figure. (*b*) Analyses of Diaph alignment processed by fast-evolving position removal (FPR). We repeated ultrafast bootstrap analyses using IQ-TREE 1.6.12 on the Diaph alignment after excluding no position, the top 20, 40, 60 and 80% fastest-evolving positions. The plots in purple, green, blue, and red indicate the ultrafast bootstrap support values (UFBPs) for the monophyly of Pancryptista, the monophyly of Archaeplastida, CAM clade, and the union of Rhodophyta and Pancryptista, respectively. (*c*–*k*) Analyses of the alignments generated by random gene sampling (RGS). We sampled 50, 100, 150 and 200 genes randomly from the 319 genes in the Diaph alignment, concatenated into ‘rs50g,’ ‘rs100g,’ ‘rs150g’ and ‘rs200g’ alignments, and subjected to ultrafast bootstrap analyses using IQ-TREE 1.6.12. We presented the UFBPs for CAM clade (i.e. the sister relationship between Pancryptista and Archaeplastida), the monophyly of Archaeplastida, and the monophyly of Pancryptista as box-and-whisker plots (*c*), (*f*) and (*i*), respectively. The above-mentioned analyses were repeated after *Rhodelphis* spp. or *M. maris* were excluded from the alignments alternatively. The UFBPs from the analyses excluding *Rhodelphis* spp. and those from the analyses excluding *M. maris* are presented in (*d*), (*g*) and (*j*), and (*e*), (*h*) and (*k*), respectively. The UFBPs shown in the plots described above are summarized in electronic supplementary material, table S3.

The Diaph alignment, which was generated by excluding 22 taxa from the GlobE alignment, was analysed to explore the impact of *M. maris* on the phylogenetic relationship among the major lineages in Diaphoretickes. Twenty-one out of the 22 taxa excluded from the GlobE alignment were not a member of Diaphoretickes—most of Opisthokonta, all discobids, *Paratrimastix pyriformis*, *Nutomonas longa,* and *Malawimonas jakobiformis*. We excluded a single member of Diaphoretickes, Picozoa sp., from the Diaph alignment due to its instability in the GlobE phylogeny, which likely stemmed from low site coverage in the GlobE alignment (figure 1). The phylogenetic relationship among the major Diaphoretickes lineages/species inferred from the Diaph alignment (e.g. the basal position of *M. maris* to the Cryptista clade) was essentially the same as that inferred from the GlobE alignment (electronic supplementary material, figures S1 and S2*a*). We here focus on the monophyly of Archaeplastida and the sister relationship between Archaeplastida and Pancryptista, both of which were fully supported in the ML and Bayesian analyses of the Diaph alignment (figure 2*a*).

Neither monophyly of Archaeplastida nor sister relationship between Archaeplastida and Cryptista has been unambiguously recovered. For instance, Gawryluk *et al*. [26] conducted analyses of an alignment comprising 254 genes but could not settle the relationship among Chloroplastida, Glaucophyta, and Rhodophyta plus Rhodelphidia. The ML analysis of their 254-gene alignment put Cryptista within the three lineages of Archaeplastida, albeit Bayesian analysis of the same alignment recovered both monophyly of Archaeplastida and sister relationship between Archaeplastida and Cryptista. In another phylogenomic study, the monophyly of Archaeplastida was not reconstructed in either ML or Bayesian analysis of an alignment comprising 248 genes, as Cryptista was tied with Rhodophyta [24]. Irisarri *et al*. [50] recently analysed a 311-gene alignment and demonstrated that taxon sampling, selection of alignment positions, and substitution models for phylogeny are critical to recovering the monophyly of Archaeplastida.

By contrast to the pioneering phylogenomic studies (see above), both monophyly of Archaeplastida and the sister relationship between Pancryptista and Archaeplastida were reconstructed from the Diaph alignment with full statistical support by both ML and Bayesian methods (figure 2*a*). Significantly, FPR analysis on the Diaph alignment appeared to have little impact on the UFBPs for the two nodes of interest (figure 2*b*). Both monophyly of Archaeplastida and sister relationship between Pancryptista and Archaeplastida received full or nearly full UFBPs until the top 60% fastest-evolving positions were removed (purple and green lines, respectively, in figure 2*b*). We also analysed rs50g, rs100g, rs150g and rs200g alignments generated from the Diaph alignment (figure 2*c*,*f*,*i*; see also electronic supplementary material, table S3 for the details). The results from RGS analyses clearly indicate that the UFBPs for the two nodes of interest (and that for the monophyly of Pancryptista) increased in proportion to the number of genes considered. We here conclude that Archaeplastida is a genuine clade as demonstrated by Irisarri *et al*. [50], and Pancryptista is the closest relative of Archaeplastida and thus propose the sister relationship between Archaeplastida and Pancryptista as ‘CAM’ clade. The proposed name is an acronym derived from the first letters of Cryptista, Archaeplastida and *Microheliella*.

It is significant to note that the monophyly of Archaeplastida and the sister relationship between Archaeplastida and Cryptista was recovered by a 311-gene phylogeny prior to the *M. maris* data are available [7,50]. Thus, we decided to evaluate systematically how the inclusion of *M. maris*, as well as that of Rhodelphidia, contributed to the recovery of the monophyly of Archaeplastida and the sister relationship between Archaeplastida and Pancryptista/Cryptista. We re-analysed rs50g, rs100g, rs150g and rs200g alignments generated from the Diaph alignment after excluding Rhodelphidia or *M. maris* (figure 2*d*,*e*,*g*,*h*,*j*,*k*; see also electronic supplementary material, table S4 for the details). In the analyses of rs50g and rs100g alignments, the removal of Rhodelphidia/*M. maris* lowered the overall distributions of the UFBPs for the monophyly of Archaeplastida and sister relationship between Archaeplastida and Pancryptista/Cryptista (figure 2*d*,*e*,*g*,*h*). However, most of the UFBPs for the two groupings of interest in the analyses of rs200g alignments were around or greater than 90% (figure 2*d*,*e*,*g*,*h*; electronic supplementary material, table S4). Likewise, the overall distribution of the UFBPs for the monophyly of Pancryptista was apparently lowered in the analyses of rs50g, rs100g and rs150g alignments in the absence of Rhodelphidia (figure 2*j*). After *M. maris* was excluded, the monophyly of Cryptista was constantly recovered with full UFBPs, except a UFBP of 21.5% obtained in the analyses of a single rs50g alignment (figure 2*k*; electronic supplementary material, table S4). The removal of Rhodelphidia or *M. maris* appeared to possess a moderate but apparent impact on the monophyly of Archaeplastida and sister relationship between Archaeplastida and Pancryptista/Cryptista in the alignments comprising 100 or fewer genes, albeit such impact can be overcome by an increment of the alignment size.

### 3.3. On the artefactual grouping of Rhodophyta and Cryptophyceae

The phylogenetic analyses described above indicated that taxon sampling is a key to recover the monophyly of Archaeplastida and the sister relationship between Archaeplastida and Pancryptista (CAM clade) with confidence. Then, why did phylogenomic analyses, in which either or both of Rhodelphidia and *M. maris* were absent, often failed to recover the monophyly of Archaeplastida? For instance, a recent phylogenomic study [24], which considered neither Rhodelphidia nor *M. maris*, grouped Rhodophyta and Cryptista together instead of recovering the monophyly of Archaeplastida.

**Figure 3.**
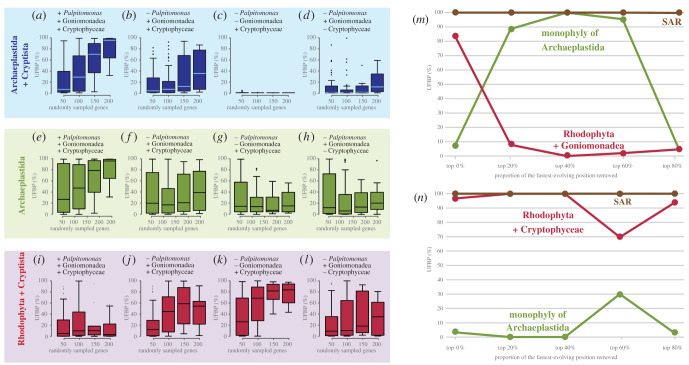
Analyses assessing the phylogenetic affinity of Rhodophyta to Cryptophyceae and/or Goniomonadea. (*a*–*l*) Analyses of the alignments generated by random gene sampling (RGS). We excluded both *Rhodelphis* spp. and *Microheliella maris* from the ‘rs50g,’ ‘rs100g,’ ‘rs150g’ and ‘rs200g’ alignments, which were generated from the Diaph alignments (see Methods for the detail) and then subjected to the ultrafast bootstrap analyses using IQ-TREE 1.6.12. The ultrafast support values (UFBPs) for the sister relationship between Archaeplastida and Cryptista, the monophyly of Archaeplastida, and the union of Rhodophyta and Cryptista are presented as box-and-whisker plots (*a*), (*e*) and (*i*), respectively. The ultrafast bootstrap analyses on the rs50g, rs100g, rs150g and rs200g alignments were repeated after further exclusion of *Palpitomonas bilix* (*b*, *f* and *j*), *P. bilix* and Goniomonadea (*c*, *g* and *k*), and *P. bilix* and Cryptophyceae (*d*, *h* and *l*). The UFBPs shown in the plots described above are summarized in electronic supplementary material, table S4. (*m*,*n*) Analyses of the alignments processed by fast-evolving position removal (FPR). We modified the Diaph alignment in two ways, (i) the exclusion of *Rhodelphis* spp., *M. maris*, *P. bilix*, and Cryptophyceae and (ii) that of *Rhodelphis* spp., *M. maris*, *P. bilix* and Goniomonadea. The two modified Diaph alignments were processed by FPR and further subjected to ultrafast bootstrap analyses. We plotted the UFBPs for the monophyly of the SAR clade (brown), those for the monophyly of Archaeplastida (green), and those for the uniting of Rhodophyta and Goniomonadea/Cryptophyceae (red).

The absence of both Rhodelphidia and *M. maris* had a greater impact on the UFBP for the monophyly of Archaeplastida and that for the sister relationship between Archaeplastida and Cryptista than the absence of either of the two lineages/species. Regardless of the number of randomly sampled genes, the distributions of the UFBPs for the two groupings of interest tend to be lower than the corresponding values calculated from the analyses excluding either Rhodelphidia or *M. maris* (compare figure 2*d*,*e* with figure 3*a*, and figure 2*g*,*h* with figure 3*e*; see also electronic supplementary material, table S3 for the details). Interestingly, the faint affinity between Rhodophyta and Cryptista became detectable in the absence of Rhodelphidia and *M. maris* (figure 3*i*). After *P. bilix* was additionally excluded (Cryptista was represented by Goniomonadea and Cryptophyceae), both UFBP for the monophyly of Archaeplastida and that for the sister relationship between Archaeplastida and Cryptista were further lowered (figure 3*b*,*f*). In stark contrast, the exclusion of *P. bilix* enhanced the affinity between Rhodophyta and Cryptista (figure 3*j*). These results clearly indicated that, in the absence of Rhodelphidia and *M. maris*, *P. bilix* possesses a significant impact on the recovery of the monophyly of Archaeplastida by excluding Cryptista.

We further analysed the alignments in which Cryptista was represented solely by Cryptophyceae or Goniomonadea. The most drastic results were obtained from the analyses of the alignments in which Cryptophyceae was the sole representatives of Cryptista (figure 3*c*,*g*,*k*; see also electronic supplementary material, table S4 for the details). After the exclusion of the non-photosynthetic lineages in CAM clade (i.e. Rhodelphidia, *M. maris*, *P. bilix* and Goniomonadea), the union of Cryptista (i.e. Cryptophyceae) and Rhodophyta appeared to dominate over the monophyly of Archaeplastida, particularly in the analyses of larger-size alignments (figure 3*g*,*k*). The analyses of rs200g alignments recovered the union of Cryptista and Rhodophyta with UFBPs ranging from 43.2 to 97.6% (electronic supplementary material, table S4). Of note, the decrease in UFBP support values appeared to be much more severe for the sister relationship between Archaeplastida and Cryptista than the monophyly of Archaeplastida—the UFBPs for the former grouping were 0 or nearly 0, regardless of the alignment size (figure 3*c*; electronic supplementary material, table S4). Compared to the analyses considering Cryptophyceae as the sole representative of Cryptista, we observed only the mild suppression of the monophyly of Archaeplastida and that of the sister relationship between Archaeplastida and Cryptista in the analyses of the alignments in which Cryptista was represented by Goniomonadea (figure 3*d*,*h*; electronic supplementary material, table S4). These observations coincide with the affinity between Goniomonadea and Rhodophyta being more weakly supported (figure 3*l*) than that between Cryptophyceae and Rhodophyta (figure 3*k*).

We revealed that Rhodophyta was attracted to Goniomonadea and Cryptophyceae erroneously in the ML phylogenies inferred from the alignments lacking Rhodelphidia, *M. maris* and *P. bilix* (see above). In the global eukaryotic phylogeny, Rhodelphidia interrupts the branch leading to the Rhodophyta clade. Likewise, *M. maris* and *P. bilix*, both of which are basal branches of the Pancryptista clade, break the branch leading to the clade of Cryptophyceae and Goniomonadea (i.e. Cryptomonada). Thus, the grouping of Rhodophyta and Cryptomonada is most likely the phylogenetic artefact in which the two long branches, one exposed by the absence of Rhodelphidia and the other exposed by the absence of *M. maris* and *P. bilix*, attract to each other—LBA artefact [32]. Importantly, this phylogenetic artefact could not be overcome completely in the analyses of the alignments comprising at least 200 genes (figure 3*j*–*l*). If so, we anticipated that the putative phylogenetic artefact uniting Rhodophyta and Cryptomonada was enhanced further by the exclusion of Cryptophyceae (or Goniomonadea) (figure 3*k*,*l*), as this procedure extended the branch leading to the clade of Goniomonadea (or Cryptophyceae). The exclusion of Goniomonadea (i.e. Cryptophyceae were the sole representatives of Cryptomonada) appeared to enhance the putative phylogenetic artefact much greater degree than the exclusion of Cryptophyceae (i.e. Goniomonadea were the sole representatives of Cryptomonada) (compare figure 3*c* with *d*, figure 3*g* with *h*, and figure 3*k*,*l*). These results imply that the phylogenetic artefact uniting Rhodophyta and Cryptophyceae is substantially different from that uniting Rhodophyta and Goniomonadea. Altogether, we conclude that both size and taxon sampling, particularly the sampling of the members of CAM clade, in alignments heavily matter to reconstruct the monophyly of Archaeplastida and sister relationship between Archaeplastida and Pancryptista/Cryptista with confidence.

To pursue the reason why Rhodophyta is artefactually attracted to Cryptophyceae more severely than Goniomonadea (see above), we modified the Diaph alignment by excluding Rhodelphidia and all members of Pancryptista except Goniomonadea, and the resultant alignment was then subjected to FPR analysis (figure 3*m*). In the analysis of the alignment with full positions, the union of Goniomonadea and Rhodophyta received a UFBP of greater than 80%, while the monophyly of Archaeplastida was supported by a UFBP of smaller than 10%. The analyses of the alignments after removing the top 20–60% fastest-evolving positions drastically increased the UFBP for the monophyly of Archaeplastida (89–100%; green line in figure 3*m*), while the UFBP for the grouping of Rhodophyta and Goniomonadea was reduced to less than 10% (red line in figure 3*m*). Thus, we conclude that the union of Rhodophyta and Goniomonadea is the typical LBA artefact stemming from fast-evolving positions. We repeated the same analysis described above but substituted Goniomonadea with Cryptophyceae (figure 3*n*). Unexpectedly, the union of Rhodophyta and Cryptophyceae received UFBP of 98%, 100%, 100%, 70% and 92% in the analyses after removal of top 20%, 40%, 60% and 80% fastest-evolving positions, respectively (red line in figure 3*n*). The UFBP for the monophyly of Archaeplastida was less than 10%, except the analysis after removal of the top 60% fastest-evolving positions gave a UFBP of 30% (green line in figure 3*n*). These results strongly suggest that, in terms of dependency on fast-evolving positions, the phylogenetic artefact uniting Rhodophyta and Cryptophyceae is distinct from the typical LBA artefact uniting Rhodophyta and Goniomonadea.

### 3.4. Exploring the biological perspective on the ‘signal’ uniting Rhodophyta and Cryptophyceae recovered in phylogenomic analyses

It is attractive to propose that the difference between the artefact uniting Rhodophyta and Cryptophyceae and that uniting Rhodophyta and Goniomonadea stems from the difference in lifestyle between the two closely related lineages in Cryptista. Goniomonadea is primarily heterotrophic and their nuclear genomes are free from endosymbiotic gene transfer (EGT) [51]. Indeed, the series of the phylogenetic analyses described above demonstrated that the typical LBA was sufficient to explain the union of Rhodophyta and Goniomonadea (illustrated typically by figure 3*m*). By contrast, the extant member of Cryptophyceae possesses the plastids that were traced back to a red algal endosymbiont in the common ancestor of Cryptophyceae. During the red algal endosymbiont being transformed into a host-governed plastid, a number of genes had been transferred from the endosymbiont nucleus to the host nucleus. If a phylogenomic alignment contains genes acquired from the red algal endosymbiont, such genes are the source of the phylogenetic ‘signal’ uniting Cryptophyceae and Rhodophyta. However, we selected the 319 genes, each of which showed no apparent sign of EGT in the corresponding single-gene phylogenetic analysis, for the phylogenomic analyses in this study. Additionally, we calculated the log-likelihoods (lnLs) of two identical tree topologies except for the position of Cryptophyceae—one bearing the monophyly of Archaeplastida (Tree 1) and the other bearing the grouping of Rhodophyta and Cryptophyceae (Tree 2)—for each of the 319 single-gene alignments (note that Rhodelphidia, *M. maris*, *P. bilix* and Goniomonadea were omitted from the alignments) (electronic supplementary material, figures S5a and S5b). The 319 single-gene alignments were sorted by the lnL difference between the two test trees (normalized by the alignment lengths) and the top 10 alignments, which prefer Tree 2 over Tree 1, were subjected individually to the standard ML phylogenetic analyses (electronic supplementary material, figure S6, see also electronic supplementary material, table S5 for the details). Nevertheless, we did not detect any strong phylogenetic affinity between Rhodophyta and Cryptophyceae in any of the 10 ML single-gene analyses (electronic supplementary material, figure S6, see also electronic supplementary material, table S5). These results cannot be explained by a simple scenario assuming that a subset of the ‘cryptophycean genes’ in the phylogenomic alignment was in fact acquired endosymbiotically from the red algal endosymbiont as briefly mentioned in Cavalier-Smith *et al*. [31]. We examined an additional scenario which assumes that a potentially large number of the cryptophycean nuclear genes (including those composed of the phylogenomic alignment) are the chimeras of the sequence inherited vertically beyond the red algal endosymbiosis and that acquired from the red algal endosymbiont. In each chimeric gene, the phylogenetic signal from the red algae-derived gene portion is likely insufficient to unite Rhodophyta and Cryptophyceae together in the single-gene analysis. However, when multiple chimeric genes in the cryptophycean nuclear genomes were included in a phylogenomic alignment, the phylogenetic signal from the red algae-derived gene portion becomes detectable as the union of Rhodophyta and Cryptista in the absence of Rhodelphidia and the basal branching taxa in Pancryptista, such as *M. maris* and *P. bilix*. To examine the second scenario, we additionally calculated the site-wise lnL differences between Trees 1 and 2, albeit no clear sign for the putative red algal gene fragments was detected (electronic supplementary material, figure S7, see also electronic supplementary material, table S6). Although the results described above cannot exclude the potential chimerization of the nuclear genes in Cryptophyceae completely, we have no plausible explanation for the artefactual union of Rhodophyta and Cryptophyceae now. When we clarify the principal reason why Rhodophyta and Cryptophyceae artefactually attracted to each other in phylogenomic analyses, we may unveil an as-yet-unknown commonality in genome evolution between the two separate branches in the tree of eukaryotes.

## 4. Conclusion

In this work, we successfully deepen our understanding of the early evolution of eukaryotes. The phylogenomic analyses presented here demonstrated that *M. maris* is critical to understanding the early evolution of Cryptista, as well as that of Archaeplastida. We also revealed that the deep branches of Archaeplastida and Pancryptista—Rhodelphidia, *M. maris*, *P. bilix* (although not examined in this study, Picozoa most likely possesses the equivalent impact to the above-mentioned species/lineages, too)—are critical to suppress the cryptic and severe phylogenetic ‘signal’ in cryptophycean genes.

## Data Availability

The transcriptome data of *M. maris* and *H. marina* were deposited in GenBank/EMBL/DDBJ Sequence Archive under the accession nos. DRR333359 and DRR333360. The transcriptome assemblies of *M. maris* and *H. marina*, and the phylogenetic alignments analysed in this study are available from the Dryad Digital Repository: https://doi.org/10.5061/dryad.jdfn2z3cv. The data are provided in electronic supplementary material [52].
